# Posterior myocardial infarction caused by superdominant circumflex occlusion over an absent right coronary artery: Case report and review of literature

**DOI:** 10.1097/MD.0000000000026604

**Published:** 2021-07-09

**Authors:** Seok Oh, Ju Han Kim, Min Chul Kim, Young Joon Hong, Youngkeun Ahn, Myung Ho Jeong

**Affiliations:** Department of Cardiology, Chonnam National University Hospital, Gwangju, Korea.

**Keywords:** acute myocardial infarction, percutaneous coronary intervention, right coronary artery absence

## Abstract

**Rationale:**

Congenital agenesis of the right coronary artery (CARCA) initially presenting as acute myocardial infarction (AMI) due to total occlusion is a rare clinical condition that can lead to severe complications, including death. We report a case of successful percutaneous coronary intervention (PCI) in a patient with this condition.

**Patient concerns:**

A 57-year-old man was admitted to our center with chest pain that had occurred several hours prior. Since he was initially diagnosed with AMI with ST-segment elevation, we promptly commenced coronary angiography (CAG).

**Diagnosis:**

CAG revealed the absence of a right coronary artery (RCA). In the left coronary cusp area, the left circumflex coronary artery (LCX) was occluded totally.

**Interventions:**

We performed PCI for total occlusion of the proximal part of the LCX. Follow-up CAG showed a superdominant branch of the LCX, sprouting into the RCA territory.

**Outcomes:**

The patient was discharged uneventfully after successful PCI.

**Lessons:**

CARCA with AMI, which is an extremely unusual case, can be fatal; however, PCI seems to be an effective treatment option.

## 1 Introduction

A single coronary artery (SCA) is a rare congenital malformation in which only one epicardial coronary artery arises from the aortic root by the SCA ostium, supplying the entire area of the heart.^[1]^ This anomaly accounts for 0.014% to 0.066% of the population who undergo diagnostic coronary angiography (CAG).^[2–5]^ Congenital agenesis of the right coronary artery (CARCA) is a very uncommon type of SCA, in which a single left coronary artery (LCA) exists, with an absent ostium of the RCA; rather, the RCA originates from any portion of the LCA. To date, fewer than 80 cases of this malformation have been reported in the literature. Although this disorder is usually asymptomatic and considered benign, a small proportion of patients with CARCA develop life-threatening clinical situations, including myocardial infarction (MI), stroke, and sudden cardiac arrest.^[2,4,6]^ In some reported cases, percutaneous coronary intervention (PCI) was performed.^[7–10]^

Herein, we describe an extremely unusual case of a patient with CARCA and MI who was successfully treated with PCI for total occlusion of the left circumflex coronary artery (LCX). Our study was conducted according to the ethical standard of the Declaration of Helsinki. The patient's clinical information and data were all available in the electronic medical records of Chonnam National University Hospital. This study was exempted from the review of the institutional review board (IRB) of our hospital (IRB exempt number: CNUH-EXP-2021–126) and the informed consent was obtained from the patient.

## 2 Case report

A 57-year-old man was brought to our tertiary center with anginal pain as the chief complaint for several hours, which tended to be squeezing, without radiation. This anginal symptom gradually worsened, prompting him to visit our hospital for diagnosis and management.

**Figure 1:**
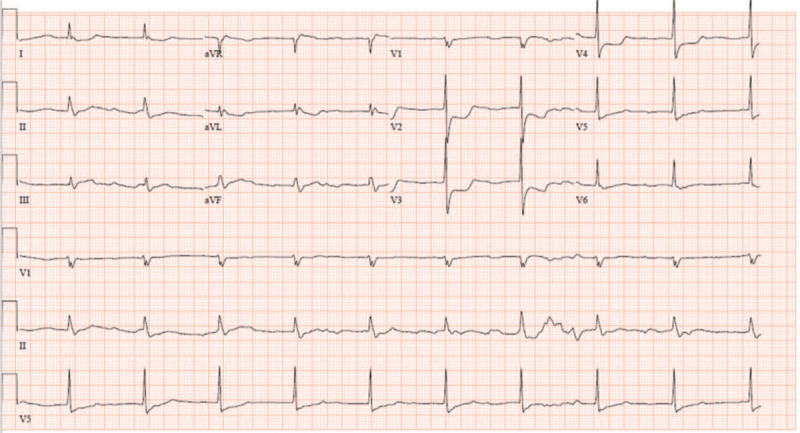
The initial electrocardiogram demonstrates ST-segment elevation in aVR and ST-segment depression in precordial leads V2 to V4.

**Figure 2:**
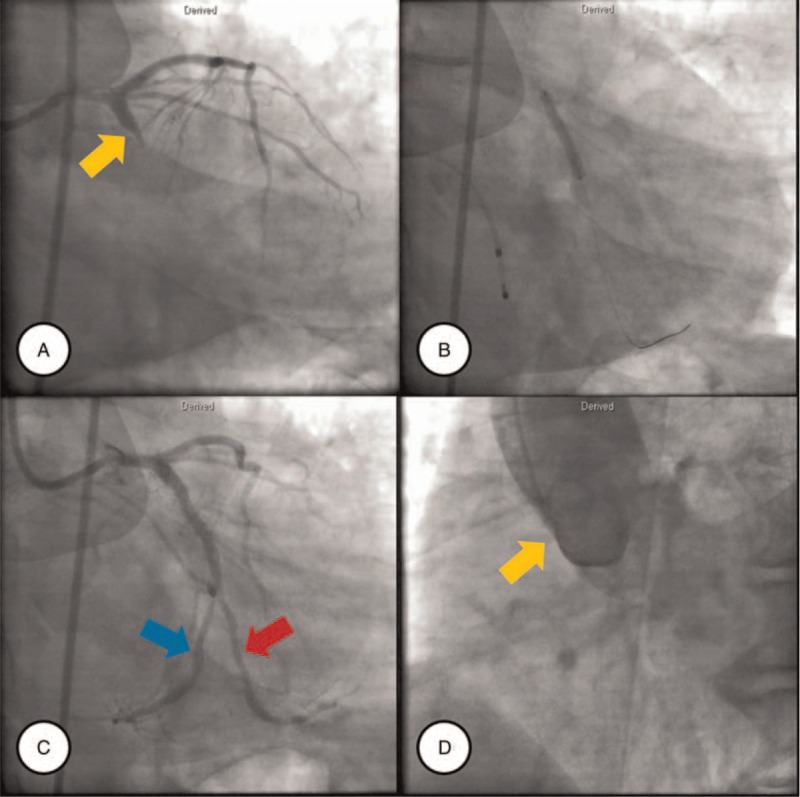
A, CAG demonstrates a total occlusion in the proximal shaft of the LCX (yellow arrows). B, C, After deploying a sirolimus-eluting coronary stent system (BioMime, Meril Life) in the occlusive lesion, the interim CAG shows marked improvement in the distal flow and 2 big branches from the LCX, among which one (red arrow) follows the LCX distribution course, and another one (blue arrow) followed the RCA distribution course. D, Aortography demonstrates the lack of a separate ostium of the RCA within the right coronary cusp. CAG = coronary angiography, LCX = left circumflex coronary artery, RCA = right coronary artery.

The patient had no documented medical history. He was a current smoker, with approximately 30 pack-years of smoking. His father received a coronary artery bypass graft (CABG) due to MI. On physical examination, his temperature was 36.1°C, heart rate was 60/min, respiratory rate was 28/min, and blood pressure was 70/40 mm Hg. Crackle sounds were heard in both chest areas, suggesting concomitant pulmonary congestion. His heart examination findings were normal. On the initial electrocardiogram, the ST-segment was elevated in aVR, but depressed in precordial leads V2 to V4 (Fig. 1). A right-sided electrocardiogram was also obtained to detect right ventricular MI, which demonstrated ST-segment elevation from V7 to V9 (Supplementary Fig. 1), indicative of MI in the right ventricular wall. His high-sensitivity troponin-T level was 0.020 ng/mL (reference range, 0–0.014 ng/mL) and creatine kinase isoenzyme level was 2.08 ng/mL (reference range, 0–5 ng/mL). Because a diagnosis of ST-elevation MI was clinically suspected, with concomitant hemodynamic instability, loading doses of aspirin and ticagrelor were administered, and emergent CAG was performed. CAG via the right transfemoral artery demonstrated total occlusion of the proximal portion of the LCX (Fig. 2A). There was a protruding branch from the left coronary cusp into the conus branch. The RCA was invisible from the orifice at the aortic root.

After consent from all family members was obtained, PCI for LCX total occlusion was performed. Initially, an EBU 3.5 catheter was engaged into the LCA orifice. A guidewire (SION BLUE, Asahi) was then advanced into the total occlusive lesion of the LCX. A 2.5 × 15-mm balloon was delivered along this guidewire into the diseased portion of the LCX and predilated to 6 atm (Fig. 2B). The distal flow of the LCX was fully restored (from grade 0 to grade III thrombolysis in myocardial infarction flow), but the stenosed portion was remnant. However, the LCX vasculature stretched into 2 large branches. While one branch followed the normal LCX course distribution in the anterograde direction, the other branch followed the RCA distribution course in the retrograde direction, which meant that the entire RCA myocardium was dominated by a superdominant LCX (Fig. 2C). A 3.00–24-mm sirolimus-eluting coronary stent system (BioMime, Meril Life) was implanted in the remnant stenosed region. After that, a 3.5–15-mm noncompliant balloon was postdilated with 18 atm. The final CAG showed good distal antegrade flow (thrombolysis in myocardial infarction flow grade III) into the LCX without significant stenosis (see Supplementary Video 1, which demonstrates the superdominant LCX stretching into the RCA territory). After PCI, an aortogram was additionally performed to determine the absence of the RCA. There was no definite evidence for the presence of RCA vasculature, indicating a CARCA (Fig. 2D).

**Figure 3:**
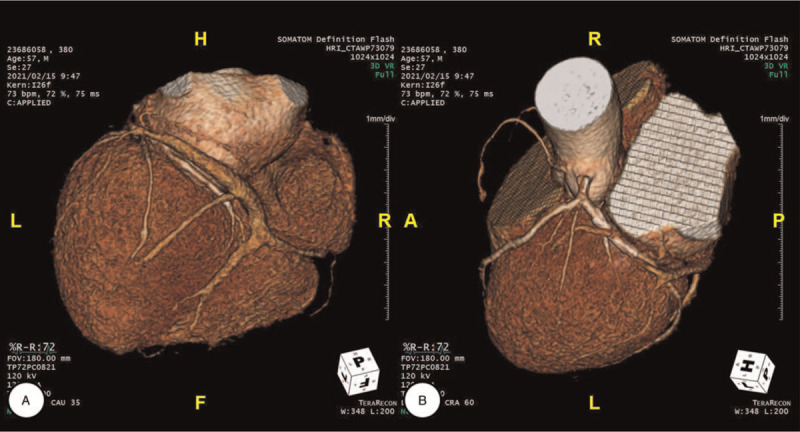
A, B, Coronary CTA demonstrates a normal LAD course, without calcified or noncalcified plaque formation. However, the ostium of the RCA is absent, except for only a small protruding branch from the LCC into the conus branch. Instead, a large branch originating from the LCX passes through the atrioventricular groove, supplying the RCA distribution. CTA = computed tomography angiography, LAD = left anterior descending coronary artery, LCC = left coronary cusp, LCX = left circumflex coronary artery, RCA = right coronary artery.

Afterward, the patient was transferred to the intensive care unit for close hemodynamic monitoring and was administered optimal medical therapy, including antiplatelet therapy (aspirin and ticagrelor), high-intensity statin (atorvastatin, 40 mg/d), beta-blocker (bisoprolol, 1.25 mg/d), and angiotensin receptor blocker II (valsartan, 40 mg/d). Several days later, he was moved to the general ward and underwent computed tomography angiography (CTA) of the coronary artery using a 2 × 128-slice dual-source dual-energy scanner (SOMATOM Definition Flash, Siemens Healthcare, Erlangen, Germany). Image acquisition was performed to secure a volume from the level of the tracheal bifurcation through the diaphragm using prospective electrocardiogram gating according to the heartbeat. CTA revealed a normal left anterior descending coronary artery (LAD) course without calcified or noncalcified plaque formation. However, a RCA ostium was not visible at the aortic root, except for a small conus branch from the left coronary cusp. Instead, a large branch arising from the LCX and passing through the atrioventricular groove sprouted into the RCA distribution, supplying this area (Fig. 3, Supplementary Video 2). Since the complete absence of the RCA was identified, the diagnosis of CARCA was reconfirmed.

## 3 Discussion

The developmental process of epicardial coronary arteries is subdivided into 2 stages: immature vascular plexus formation and subsequent remodeling into a mature vascular bed. This vascular network migrates into the myocardial layer; the epicardial cells then undergo epithelial–mesenchymal transition to form smooth muscle cells and fibroblasts. These attach to the aorta to initiate blood flow, resulting in the mature cardiac coronary artery system.^[11,12]^ Anomalous alterations in the coronary artery are due to problems in this embryological process. SCA accounts for a small proportion of the broad spectrum of coronary anomalies. CARCA, a special subgroup of SCA, was first mentioned in 1946 by White and Edwards.^[13]^

The angiographic classification of CARCA was first proposed by Lipton et al in 1979,^[3]^ and this classification was modified by Yamanaka et al in 1990.^[2]^ According to this classification, CARCA is subdivided into 2 types: the L-I type, and the L-II type. The L-I type involves the distal extension of the LCX or left anterior descending coronary artery that controls the myocardium of the RCA distribution. The L-II type consists of the branch derived from the proximal portion of the LCA that controls the myocardium of the RCA distribution. The current case was compatible with the L-I type of the Lipton classification of coronary anomalies.

CAG is currently the gold standard for the diagnosis of CARCA. Echocardiography is a useful cardiovascular modality for differentiating other structural abnormalities combined with SCA. Additionally, coronary CTA is a noninvasive modality and is extensively used in clinical settings.^[14–16]^ It provides three-dimensional information on epicardial coronary arteries and cardiac chambers.^[17]^ Coronary CTA does not suffer any operational difficulty due to anatomical malformations, and has a high level of diagnostic accuracy. In addition, coronary CTA can be used to detect other anatomical abnormalities simultaneously. Therefore, combining these imaging modalities can produce a more accurate assessment of the epicardial coronary arteries and other cardiovascular structures than a single modality.^[18]^

In patients with CARCA, a wide spectrum of clinical manifestations can be observed.^[5]^ Although it is still unknown how CARCA induces myocardial ischemia, many experts have suggested several mechanisms, such as coronary steal phenomenon and slow flow-induced ischemia due to the long travel distance of the abnormal coronary artery.^[19,20]^

In a PubMed search for cases of CARCA published from January 2011 to December 2020, a total of 38 cases were found in 34 articles (Table 1).^[7,21–29]^ Among these cases, a total of 22 patients were male, 13 were diagnosed with MI, and 15 underwent PCI or CABG. Furthermore, 29 cases showed L-I type CARCA. Thus, L-I type CARCA may be more frequent than L-II type CARCA.

**Table 1:** Clinical characteristics of cases with CARCA reported from 2011 to 2020.

Author	Year	Number of cases	Sex	Age (years)	AMI	PCI or CABG	Diagnostic modalities	Origin of RCA	Lipton classification
Gitsioudis, et al	2011	1	Female	66	0	0	CAG+CTA	LAD	L-I pattern
Kalyani, et al	2011	1	Male	30	0	N/A	Autopsy	LCX	L-I pattern
Saremi, et al	2011	1	Male	46	0	0	CAG+CTA	LAD	L-II pattern
Sonmez, et al	2011	1	Male	57	0	0	CAG	LCX	L-I pattern
Zhu, et al	2011	3	Female	77	0	1	CAG+CTA	LAD	L-II pattern
			Female	72	0	1	CAG+CTA	LAD	L-II pattern
			Male	77	0	1	CAG	LAD	L-II pattern
Blaschke, et al	2012	1	Female	32	0	0	CAG+CTA	LAD	L-I pattern
Liu, et al	2012	1	Female	69	0	0	CAG+CTA	LCX	L-I pattern
Ma, et al	2012	1	Male	39	1	1	CAG	LCX	L-I pattern
Morimoto, et al	2012	1	Female	89	0	0	CTA	LCX	L-I pattern
Nasir, et al	2012	1	Male	51	1	0	CAG+CTA	LCX	L-I pattern
Turfan, et al	2012	1	Male	58	0	0	CAG+CTA	LCX	L-I pattern
Almansori, et al	2013	1	Male	27	0	0	CAG	LCX	L-I pattern
Devidutta, et al	2013	1	Female	52	0	1	CAG+CTA	LCX	L-I pattern
Phasalkar, et al	2013	1	Female	3	0	0	CAG+CTA	LAD	L-I pattern
Puerbehi, et al	2013	1	Male	47	1	1	CAG	LCX	L-I pattern
Toyono, et al	2013	1	Female	2	0	0	CAG	LAD	L-II pattern
de Agustin, et al	2014	1	Male	40	0	0	CTA	LCX	L-I pattern
Mishra, et al	2014	1	Male	55	0	1	CAG+CTA	LCX	L-I pattern
Pourafkari, et al	2014	1	Male	44	0	1	CAG	LCX	L-I pattern
Gupta, et al	2015	1	Male	62	0	0	CAG	LCX	L-I pattern
Kim, et al	2015	1	Female	13	1	0	CAG+CTA	LCX	L-I pattern
Kus, et al	2015	1	Female	49	0	0	CAG+CTA	LCX	L-I pattern
Turkmen, et al	2015	1	Male	22	0	N/A	Autopsy	LCX	L-I pattern
Jung, et al	2016	1	Female	73	1	1	CAG+CTA	LMCA	L-II pattern
Hansen, et al	2017	1	Male	70	1	0	CAG	LCX	L-I pattern
Witkowska, et al	2017	1	Male	40	1	0	CAG+CTA	LCX	L-I pattern
Elbadawi, et al	2018	1	Female	56	0	0	CAG	LCX	L-I pattern
Lee, et al	2018	1	Male	43	0	0	CTA	LCX	L-I pattern
Yan, et al	2018	1	Male	63	1	0	CAG+CTA	LCX	L-I pattern
Zoltowska, et al	2018	1	Male	45	0	1	CAG	LMCA	L-II pattern
da Silva Matte, et al	2019	1	Male	80	1	1	CAG	LCX	L-I pattern
Iftikhar, et al	2019	1	Male	45	1	1	CAG	LCX	L-I pattern
Chen, et al	2020	2	Female	54	1	1	CAG	LAD	L-II pattern
			Male	67	1	1	CAG	LAD	L-I pattern
Forte, et al	2020	1	Female	45	0	0	CT	LCX	L-I pattern
Liu, et al	2020	1	Female	53	1	1	CAG+CTA	LAD	L-II pattern

AMI = acute myocardial infarction, CABG = coronary artery bypass graft, CAG = coronary angiography, CARCA = congenital agenesis of the right coronary artery, CTA = computed tomography angiography, LAD = left anterior descending coronary artery, LCX = left circumflex coronary artery, LMCA = left main coronary artery, PCI = percutaneous coronary intervention, RCA = right coronary artery.

Based on this literature review, several reported cases of successful PCI for MI in patients with L-I type CARCA.^[7,9,10,27,28]^ However, there are very few cases of PCI for total occlusion of the proximal LCA in patients with this malformation.^[7]^ This is the first reported case of PCI for total occlusion of the proximal LCX in a patient with L-I type CARCA confirmed on both CAG and coronary CTA. Since CARCA is a very rare condition, various modalities should be applied to discriminate it from similar other cardiovascular malformations.

## 4 Conclusion

CARCA is a very rare type of SCA that can result in various clinical manifestations; it can be life-threatening in rare cases. We report a rare case of successful PCI for total occlusion of a superdominant LCX supplying the RCA distribution in a patient with CARCA.

## Acknowledgments

This work was supported by the Chonnam National University Hospital Biomedical Research Institute (BCRI-21054).

## Author contributions

**Data curation:** Seok Oh, Min Chul Kim.

**Methodology:** Seok Oh, Ju Han Kim, Min Chul Kim.

**Writing – original draft:** Seok Oh.

**Writing – review & editing:** Seok Oh, Ju Han Kim, Young Joon Hong, Youngkeun Ahn, Myung Ho Jeong.

## Supplementary Material

Supplemental Digital Content

## Supplementary Material

Supplemental Digital Content

## Supplementary Material

Supplemental Digital Content
