# Supplementary material for: Posterior myocardial infarction caused by superdominant circumflex occlusion over an absent right coronary artery: Case report and review of literature
Source: Medicine (Baltimore). 2021 Jul 9;100(27):e26604. doi: 10.1097/MD.0000000000026604 (PMC8270598; doi:10.1097/MD.0000000000026604)

**Supplementary Fig. 1.** Right-sided electrocardiogram shows ST-segment elevation in V7 to V9, suggesting posterior wall myocardial infarction.


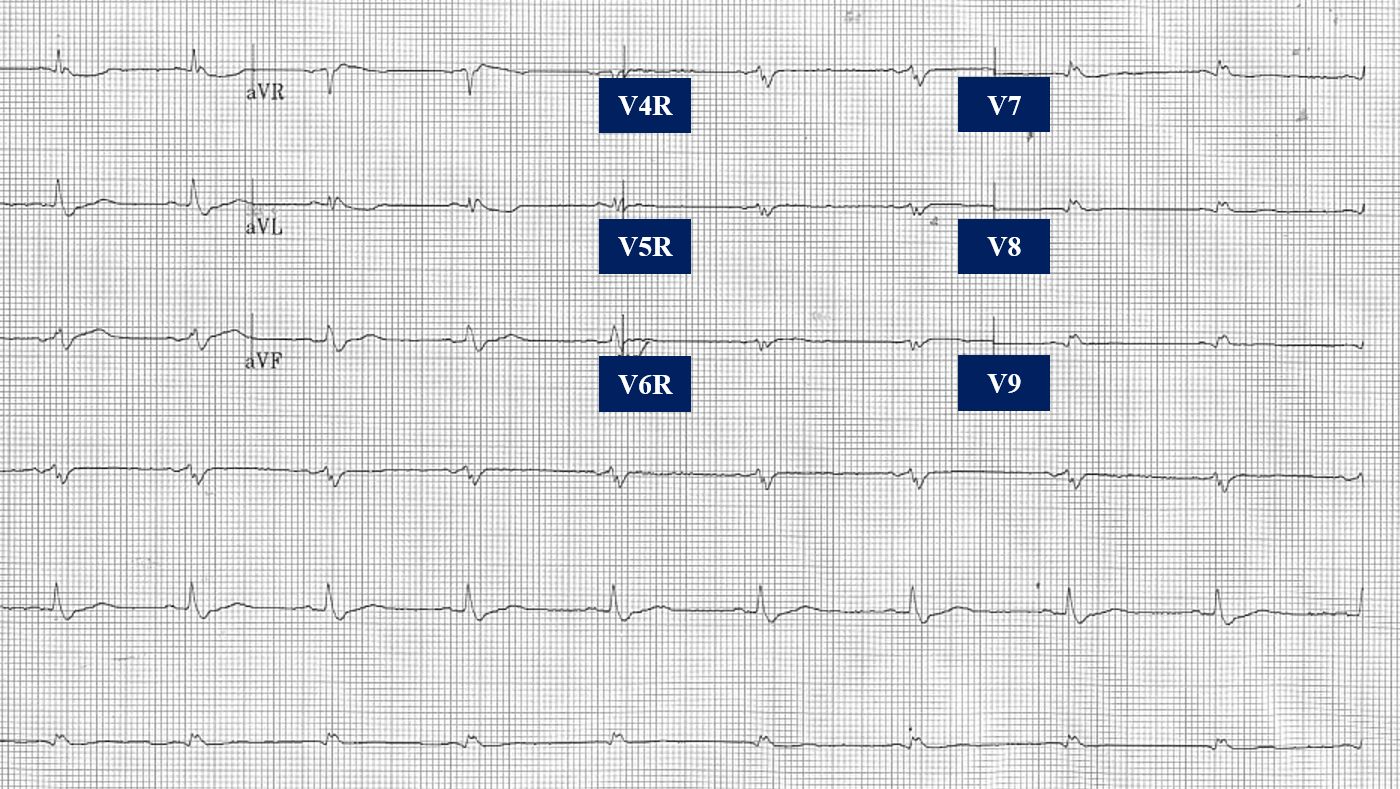

Supplement: Supplemental Digital Content [file medi-100-e26604-s001.doc]
